# Protein Kinase A Binds and Activates Heat Shock Factor 1

**DOI:** 10.1371/journal.pone.0013830

**Published:** 2010-11-09

**Authors:** Ayesha Murshid, Shiuh-Dih Chou, Thomas Prince, Yue Zhang, Ajit Bharti, Stuart K. Calderwood

**Affiliations:** 1 Molecular and Cellular Radiation Oncology, Beth Israel Deaconess Medical Center, Harvard Medical School, Boston, Massachusetts, United States of America; 2 Stress Response Center, Boston University Medical Center, Boston, Massachusetts, United States of America; Roswell Park Cancer Institute, United States of America

## Abstract

**Background:**

Many inducible transcription factors are regulated through batteries of posttranslational modifications that couple their activity to inducing stimuli. We have studied such regulation of Heat Shock Factor 1 (HSF1), a key protein in control of the heat shock response, and a participant in carcinogenisis, neurological health and aging. As the mechanisms involved in the intracellular regulation of HSF1 in good health and its dysregulation in disease are still incomplete we are investigating the role of posttranslational modifications in such regulation.

**Methodology/Principal Findings:**

In a proteomic study of HSF1 binding partners, we have discovered its association with the pleiotropic protein kinase A (PKA). HSF1 binds avidly to the catalytic subunit of PKA, (PKAcα) and becomes phosphorylated on a novel serine phosphorylation site within its central regulatory domain (serine 320 or S320), both *in vitro* and *in vivo*. Intracellular PKAcα levels and phosphorylation of HSF1 at S320 were both required for HSF1 to be localized to the nucleus, bind to response elements in the promoter of an HSF1 target gene (*hsp70.1*) and activate *hsp70.1* after stress. Reduction in PKAcα levels by small hairpin RNA led to HSF1 exclusion from the nucleus, its exodus from the *hsp70.1* promoter and decreased *hsp70.1* transcription. Likewise, null mutation of HSF1 at S320 by alanine substitution for serine led to an HSF1 species excluded from the nucleus and deficient in *hsp70.1* activation.

**Conclusions:**

These findings of PKA regulation of HSF1 through S320 phosphorylation add to our knowledge of the signaling networks converging on this factor and may contribute to elucidating its complex roles in the stress response and understanding HSF1 dysregulation in disease.

## Introduction

HSF1 is a primary regulator of the heat shock response and a factor in a number of human pathologies including cancer and neurodegenerative diseases [1], [2], [3], [4], [5]. Curiously, although both diseases are associated with advancing age, HSF1 loses activity in the progression of neurodegenerative diseases while being activated in cancer [1], [2], [6]. It would seem apparent therefore that understanding the molecular basis of HSF1 up- and down-regulation in disease would provide valuable insights.

HSF1 belongs to the multi-gene HSF family present in all eukaryotes [7]. Initial studies were carried out on the single HSF gene of the yeast *S. cereviseae* [8]. These studies indicated that, unique among transcription factors HSF undergoes trimer formation on activation and that such oligomerization governs binding to the heat shock elements (HSE) on the promoters of heat shock protein (HSP) genes [9], [10]. The findings in yeast were confirmed in mammalian cells in which trimerization was shown to be a requirement for binding to HSP promoters [7]. Another unusual feature associated with HSF is that trimerization and binding to HSE can be dissociated from *tran*s-activation in studies carried out both *in vitro* and *in vivo*; DNA binding alone is evidently insufficient to drive transcription and other, binding independent processes are involved [11], [12], [13], [14]. Early studies suggested that these may include posttranslational modification of HSF1 [10], [13], [15]. Indeed yeast HSF and mammalian HSF1 appear to undergo heavy phosphorylation on serine and threonine residues when activated [10], [15], [16]. In addition intracellular HSF1 undergoes other modifications such as sumoylation and acetylation after stress [17]. Alterations in HSF1 phosphorylation appear to be important in the “second step” of HSF1 activation and stress and *tran*s-activation of HSP genes can be inhibited by kinase inhibitors, while inactive HSF1 trimers can be rendered active *in vivo* by exposure to phosphatase inhibitors [14]. The sites of HSF1 phosphorylation have been studied by phosphopeptide mapping and a partial list of such sites exists. HSF1 is known to be phosphorylated on serines residues at 121, 230, 303, 307, 326, 363 [16], [18], [19], [20], [21], [22], [23]. The role of these sites in HSP transcription have been attributed mainly by point mutation studies and these experiments suggest that phosphorylation of serine 121, 303, 307, or 363 can inhibit HSP transcription [16], [18], [24], [25]. S230 and S326 are the only currently known phosphorylation sites associated with stimulation of transcription by HSF1. In addition, the regulatory mechanisms through which these posttranslational modifications are converted into intracellular functions are not clear [16], [18], [24]. The inhibitory modifications at serines 303, 307 and 363 have each been attributed to accelerated nuclear export [24], [26]. This effect has, in the case of serines 303 and 307 been attributed to recruitment of 14-3-3 to Phospho-S303, S307-HSF1 and stimulation of nuclear export through a pathway involving nuclear export protein CRM1/exportin1 [24]. In addition, S303 phosphorylation has been shown to lead to a secondary posttranslational modification, HSF1 sumoylation at lysine 298 [27]. Another curious aspect of HSF1 regulation during stress is that, while HSF1 phosphorylation at S303 and S307 and sumolylation at K298 are inhibitory to HSF1 function when assayed at 37°C, during heat shock these inhibitory signals are evidently inoperative and HSP transcription proceeds [28]. It seems likely that an override mechanism exists to promote rapid activation of the stress response. Persistence of inhibitory signaling may permit rapid turn off of transcription in recovery from stress after the override mechanisms of stress subside.

Much therefore remains to be learned regarding the activating roles of posttranslational modifications in HSF1 regulation, their role in stress mediated transcription and the mechanisms by which such modifications are recognized by regulatory pathways in cells and converted into altered function. We therefore began a screen of intracellular proteins that can interact with HSF1 and modify function and observed binding to a number of proteins including the catalytic subunit of 3′-5′-*cyclic adenosine monophosphate (cAMP)-dependent protein kinase* (PKAc). Protein kinase A is a versatile regulator of cell metabolism and gene transcription and consists of two main subunits, PKAc and PKA_R_ (PKA_R_ is the regulatory subunit of PKA) [29]. Before cellular activation, PKAc and PKA_R_ bind in an inactive complex that can be induced by the low molecular weight signaling molecule cAMP [30]. Cyclic AMP is a second messenger molecule generated both at the cell surface and in the cytoplasm by numerous isoforms of the enzyme adenylate cyclase [31]. Binding of cAMP to the PKA complex liberates PKAc to interact with a wide range of protein substrates, phosphorylating them on serine or threonine residues, usually within the conserved motif (RXXS/T), (where X can be any amino acid, R is arginine, S/T serine or threonine) [32]. However, a significant exception to this mechanism is found in the regulation of NFκB. PKAc binds directly to the substrate (NFκB/p65 transcription factor), phosphorylates p65 when NFκB is induced by cytokines and activates κB gene transcription independently of cAMP [20]. However, cAMP–dependent phosphorylation of p65 has also been observed [33].

We have investigated binding of the α form of PKA_C_ (PKAcα) to HSF1 and its role in *hsp70.1* transcription. PKAcα bound to HSF1 and led to phosphorylation on a novel site (serine 320). Reduction of intracellular PKAcα levels by RNA interference inhibited HSF1-S320 phosphorylation, prevented accumulation of HSF1 in the nucleus and binding to the *hsp70.1* promoter and decreased heat shock transcription. Thus PKAcα may play a novel role in activation of HSF1 and stress-induced transcription.

## Methods

### Antibodies and Reagents

Rat and Rabbit polyclonal anti-HSF1 clone 4B4/10H8 were from the Abcam and Assay Designs (Enzo Life Sciences, Inc.). Hsp70 antibody was purchased from Assay Designs (Enzo Life Sciences, Inc.). Rabbit monoclonal anti-HSF1 phospho-S320 and HSF1 phospho-S326 (Abcam), mouse monoclonal anti-HA (Covance), mouse monoclonal anti-FLAG (M2, Sigma), Rabbit polyclonal anti-PKAcα (Cell Signaling) and rabbit anti-PKA phospho-substrate (Cell Signaling) antibodies were also employed. The rabbit polyclonal PKARI antibody was from Cell Signaling and Rabbit polyclonal anti-PKRII antibody was from Santa Cruz Antibodies. The secondary antibodies were goat anti-mouse Alexa 488 (Invitrogen, USA), goat anti-rabbit Cy3 (Jackson Immuresearch Laboratories), goat anti-rabbit Cy5 (Jackson Immuresearch Laboratories), goat anti-rat Cy3 (Jackson Immuresearch Laboratories) and HRP-goat anti-mouse IgG, HRP-goat anti-rat IgG, HRP-goat anti-rabbit IgG, goat anti-Rabbit IR Dye 680 (Santa Cruz and Licor) for use in Western blotting. Heregulin, IGF-1, 17AAG, MG132, leptomycin B and t-busate were purchased from Sigma-Aldrich.

### Cells, Culture conditions and Transfection

HeLa, MCF-7, and Du145 cells were obtained from the American Type Tissue Culture Collection while HEK293FT were purchased from the Invitrogen Corporation. Cells were maintained in DMEM supplemented with 10% heat inactivated FBS, 1000 U of penicillin/streptomycin, 2 mM L-glutamine. Cells were grown in petri dishes at 37°C in a 5% CO_2_ humidified incubator. Cells were grown to 50–60% confluence on glass coverslips in six-well plates for immunofluorescence microscopy, and transfected using the Fugene TM6 reagent (Roche) according to manufacturer's instructions. All plasmids were purified using Qiagen plasmid purification kits. To generate PKAcα knockdown cells, envelope plasmid, packaging plasmid (Open Biosystems) and shRNA expressing plasmid were co-transfected into HEK293FT cells. Virus-containing medium was collected 48 and 72 hr after transfection. HeLa cells were infected by incubation with the lentivirus-containing medium and cells were treated with puromycin for selection of knockdown cells.

### DNA constructs and mutagenesis

FLAG-HSF1, HSF1-EGFP-N3, pGL3-Hsp70-LUC, pCMV-β-galactosidase constructs were prepared in-house and their construction is described in earlier publications [24], [28]. FLAG- PKAcα was made by PCR Topo-cloning (Invitrogen) the open reading frame of PKAcα (NM_002730) into pcDNA3.1 (Invitrogen). HSF1-EGFP (S320A, S320D), and FLAG-PKA (K73M) mutants were constructed using the Quickchange site-directed mutagenesis approach (Stratagene). Hairpin pLKO.1 control vector and shRNA expressing plasmids for knocking down PKAcα expression plasmids (PKAcα *kd #1* and *#2*) were obtained from Open Biosystems. Envelope plasmid (VSV-G/Pmd2.G) and packaging plasmid (pCMV-R8.74 psPAX2) were used to generate shRNA-expressing lentivirus.

### Immunoprecipitation

Cells were solubilized in ice-cold Lysis Buffer (150 mM NaCl, 1% Triton X-100, PMSF (phenyl methyl sulfonyl fluoride) protease inhibitor cocktail mix (Roche) and sodium orthovanadate). Cell lysates were probed by antibody-protein-A-sepharose cascade (GE-Healthcare), and primary and secondary precipitated proteins assayed by SDS-PAGE/immunoblot as described [34].

### Immunofluorescence and Microscopy

Cells fixed with 4% para-formaldehyde in PBS, were washed twice with 1X PBS, permeabilized using 0.1% Triton X-100 and placed in 3% normal goat serum (NGS) for 60 minutes. Cells were analyzed using a Zeiss 510 LSM confocal microscope (Carl Zeiss GmbH, Jena, Germany) using NA 1.4 63X oil immersion objective set with the pinhole set at 0.7–0.9 Airy units. GFP/Alexa 488 was visualized using 488 nm excitation and BP 505–530 emission filter. Cy3 and Cy5 were visualized using 543 nm excitation/BP 560–615 nm emission and 633 nm excitation/650 nm LP filter respectively. DAPI was visualized using 405 nm excitation and 470–500 nm emission BP filters.

### Reporter assays

Transcriptional activation of HSF1 was assayed using an *hsp70.1*-based luciferase reporter construct as described previously [35]. Results were normalized to β-galactosidase expressed from a co-transfected pCMV-LacZ plasmid [36].

### Chromatin Immunoprecipitation

ChIP assays for HSF1 association with chromatin were performed essentially as described previously [37]. ChIP was carried out with anti-HSF1 antibodies. Pre-immune IgG ChIP was used as a negative control and HSF1 binding data were normalized to this IgG control. The genomic DNA (of 1% of starting lysate/Input) was used as a positive control and pre-immune IgG mock ChIP as negative control. Amplified PCR products were first analyzed by size on agarose gel. In addition the PCR products were independently quantitated by using ABI 7300 real time PCR system and the -fold increase in ChIP-PCR products by 2-^ΔΔCT^ compared with the control (pre-immune IgG) was plotted for the respective region of *hsp70.1*. ChIP experiments and PCR amplifications were performed 3 times for each sample.

### *In vitro* kinase assay and mass spectrometric analysis of phospho-HSF1

Human HSF1 was cloned into the pGEX-2T vector and then grown up in BL21 (DE3) *E. coli* cells. The resulting GST-tagged HSF1 was isolated on a glutathione column and eluted according to the manufacturer's specifications (Pierce). For *in vitro* kinase assay, GST-HSF1 was incubated with 3 units of recombinant PKAcα, 1 µM ATP and 1X kinase assay buffer (Cell Signaling) at 37°C for 2 hr then 30 minutes at 43°C. The reaction was terminated by adding Laemmli sample buffer. For the mass spectrometric analysis, the samples were run on a 7% SDS-PAGE gel, stained with Coommassie Blue R-250 and a band greater than 85 kDa was cut out along with a “blank” band from another lane. After trypsin digestion, polypeptides were analyzed at the Taplin Mass Spectrometry Facility, Harvard Medical School, using an LTQ-Orbitrap (Thermo Electron).

### Cyclic-AMP assay

Cyclic-AMP concentrations in cell lysates were assayed according to manufacturer's instruction using an *Assay Designs* cyclic AMP Enzyme Immunoassay (EIA) kit. This is a competitive immunoassay for quantitative determination of cAMP. Levels of cAMP in HeLa cells were normalized to 100% and quantitation of cAMP was done with respect to 100% cAMP in control HeLa cells.

For experiments to determine the role of cAMP in HSF1-PKA binding, some cells were pre-treated with the adenylate cyclase inhibitor t-busate at a concentration of 200 nM for 30 minutes.

## Results

### (1) HSF1 binds PKAcα

**Figure 1:**
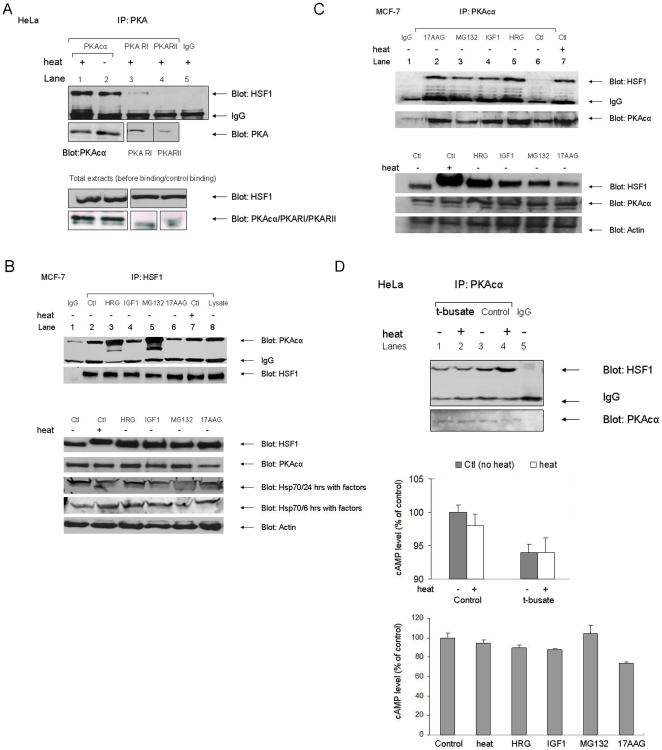
HSF1 binds to PKAcα in cells. *A)* Immunoprecipitation assays. HeLa cells were incubated for 1 hour at 37°C or 43°C before they were harvested on ice and equalized for protein concentration. Cell extracts were then probed by immunoprecipitation with either anti-PKAcα (lane 1,2) anti-PKARI (lane 3), anti-PKARII antibodies (lane 4) or rabbit IgG. Immunoprecipitates were then analyzed by 10% SDS-PAGE and immunoblot with anti-HSF1 antibodies. We have shown the IgG heavy chain to indicate the equal loading of immunoprecipitates in this experiment. The membrane was then stripped and re-probed sequentially with anti- PKAcα, PKARI and PKARII antibodies. We also analyzed the levels of HSF1, PKAcα, PKARI and PKARII in the cell lysate (prior to immunoprecipitation) and these data are shown beneath the immunoprecipitation data. Experiments were carried out in duplicate with similar findings. *B)* Immunoprecipitation assay with anti-HSF1 antibodies in MCF-7 cells. MCF-7 cells were incubated for 1 hour at 37°C or 43°C. Other cultures were simultaneously treated with growth factors (30 mM HRG or 100 ng/ml IGF-1 for 24 hr) or inhibitors (2 mM 17AAG for 24 hr, 5 µM MG-132 for 6 hr) as indicated on the figure (panels 3–6). Cell lysates were then prepared as in (A). Immunoprecipitation was performed with anti-HSF1 antibodies and immunoprecipitates later analyzed by immunoblot with anti- PKAcα antibodies. Equal amounts of immunoprecipitated proteins were loaded and immunoblotted with anti-HSF1 and anti- PKAcα antibodies. Again, we have shown the IgG heavy chain to indicate the equal loading of immunoprecipitates. In the lower panel equal amounts of protein lysates were loaded for measurement of HSF1, PKAcα, β-actin and Hsp70 in cells prior to immunoprecipitation (B, lower panel). The whole-cell lysates were analyzed by 4–15% gradient gels to demonstrate the presence of hyperphosphorylated HSF1 in the preparations more effectively. In addition we analyzed the expression of Hsp70 in cells 6 and 24 hr after the treatments to determine their effectiveness in inducing Hsp70 expression. Experiments were carried out in duplicate reproducibly. *C*) MCF-7 cells were incubated with or without heat shock for 1 hour at 43°C. Additional cell cultures were also treated with growth factors (30 µM HRG for 24 hrs, 100 ng/ml IGF-1 for 24 hrs) and inhibitors (2 µM 17AAG for 24 hrs, 5 µM MG132 for 6 hrs) at 37°C, as indicated in panels 2–5. Cells were then lysed and extracts collected and equalized for protein concentration. Immunoprecipitation was performed with anti- PKAcα antibodies prior to anti-HSF1 immunoblot. Equal amounts of protein were loaded to measure quantitatively the level of HSF1, and PKAcα. Levels of HSF1, PKAcα and β-actin in cell lysates prior to immunoprecipitation are shown in the lower panels. The experiments were carried out three times with similar findings. *D*) HSF1, adenylate cyclase and PKAcα binding. Control cells or cells given 1 hr at 43°C were treated with or without t-busate (lane 2) and then lysed on ice and immunoprecipitated with anti- PKAcα antibody. Immunoprecipitates were then fractionated by 10% SDS-PAGE and analyzed by immunoblot with anti-HSF1 antibodies. In addition, we assayed the cAMP levels in these cell lysates by EIA and these are plotted as means +/− standard deviation (right panel). Amount of cAMP was normalized to 100% of the cAMP concentration in control HeLa cells (no heat). We also measured intracellular cAMP levels (mean +/− SD) after heat shock, HRG, IGF1, MG-132 and 17-AAG treatment, using the conditions described in Fig. 1B. The cAMP levels after each of the treatments were not significantly increased compared to control. Each experiment was carried out in duplicate with similar results.

Association between HSF1 and PKAcα was first indicated in the pulldown analysis mentioned above, using GST-HSF1, HeLa cell lysates, mass spectrometry and database analysis [34] (Fig. S1). We confirmed that these proteins interact *in vivo* using immunoprecipitation of PKAcα from HeLa cell lysates by anti- PKAcα antibodies followed by immunoblot analysis with anti-HSF1 antibodies in HeLa cells (Fig. 1 A). Intracellular HSF1 was co-immunoprecipitated by these antibodies in association with PKAcα and this interaction was also observed after heat shock. We next examined whether HSF1 can bind to regulatory subunits from the PKA holoenzyme (PKA R1 or PKA RII) using co-immunoprecipiation analysis with anti-PKA-R1 and anti-PKA-RII antibodies (Fig. 1A). Trace levels of HSF1 binding to PKAR1 were observed after heat shock although we did not detect association with PKARII (Fig. 1A). However, the intracellular levels of PKA-R1 and PKA-RII were considerably lower than the concentration of PKAcα by the criterion of intensity of the immunoblot bands and preferential association may reflect this higher concentration (Fig. 1A). Nonetheless, these data are consistent with the original proteomic screen in which we found strong evidence for HSF1 binding to PKAcα but did not detect interaction of GST-HSF1 with the PKA regulatory domains.

We next carried out the inverse analysis using immunoprecipitation with anti-HSF1 antibodies followed by immunoblot with anti-PKAcα antibodies. Because HSF1 can also be activated by a number of other agents including the Hsp90 inhibitor 17AAG, the proteasome inhibitor MG-132 and growth factors such as heregulin (HRG) and insulin-like growth factor (IGF-1), we also examined the effect of these molecules on HSF1-PKAcα association. For these experiments we used MCF-7 breast carcinoma cells as these cells respond vigorously to the growth factors. We observed a significant basal level of HSF1-PKAcα binding in these cells and association was strongly increased by HRG and MG-132 (Fig. 1B). Heat shock did not cause a major increase in HSF1-PKAcα complex formation in the MCF-7 cells. However, heregulin and MG-132 led to major increases in PKA co-association (Fig. 1B). When we examined HSF1 in the lysates, we found that heat shock led to electrophoretic retardation of HSF1, consistent with stress-induced hyperphosphorylation shown previously. PKAcα levels were largely unaltered. We also measured the levels of an HSF1 product (Hsp70). Hsp70 concentrations were increased by each treatment (Fig. 1B). We then carried out the reverse procedure in MCF-7 cells, immunoprecipitating with anti-PKAcα antibodies and observed the co-precipitation of HSF1 with immunoprecipitated PKAcα (Fig. 1C). HSF1 levels were consistently increased in cells treated with HRG and HSF1-PKAcα association appeared to increase in each case (Fig. 1 B, C). This was not observed for the other treatments in which HSF1-PKAcα association was maintained after the treatments but not consistently increased (Fig. 1A–C). PKAcα levels were not consistently altered by any of the treatments and changes in levels of the kinase do not appear to influence responses to HSF1 activators (Fig. 1, A–C).

As PKA can be induced through the activation of adenylate cyclase, we examined a *scenario* in which HSF1-binding to PKAcα is regulated through the product of adenylate cyclase activity, cAMP (Fig. 1D). Indeed, using the adenylate cyclase inhibitor t-busate, we observed a reduction in HSF1-PKAcα binding, a decrease that correlates with lower cAMP levels under these conditions, suggesting a role for cAMP in the interaction. However, we did not detect an increase in intracellular cAMP levels after 1 hour of heat shock at 43°C in these cells (Fig. 1D, right panel). This could be interpreted as meaning that basal levels of cAMP are required to maintain a pool of free intracellular PKAcα capable of HSF1 binding (Fig. 1D). We next measured cAMP levels in cells treated with the other activators HRG, IGF1, 17AAG and MG-132. Once again we did not detect an increase in intracellular cAMP levels after heat shock at 43°C or with the other treatments (Fig. 1D, lower panel). However some studies have shown transient activation of adenylate cyclase, within seconds or minutes after the stress, that decay over time [38], [39]. Elevating cAMP levels with the drug forskolin in the absence of stress failed to activate HSF1-PKAcα binding (A. Murshid & SK Calderwood, unpublished data). Thus although basal cAMP may be required for HSF1-PKAcα binding, alterations in cAMP after HSF1 activation do not seem to be involved.

### (2) HSF1 is phosphorylated on serine 320 by PKAcα

**Figure 2:**
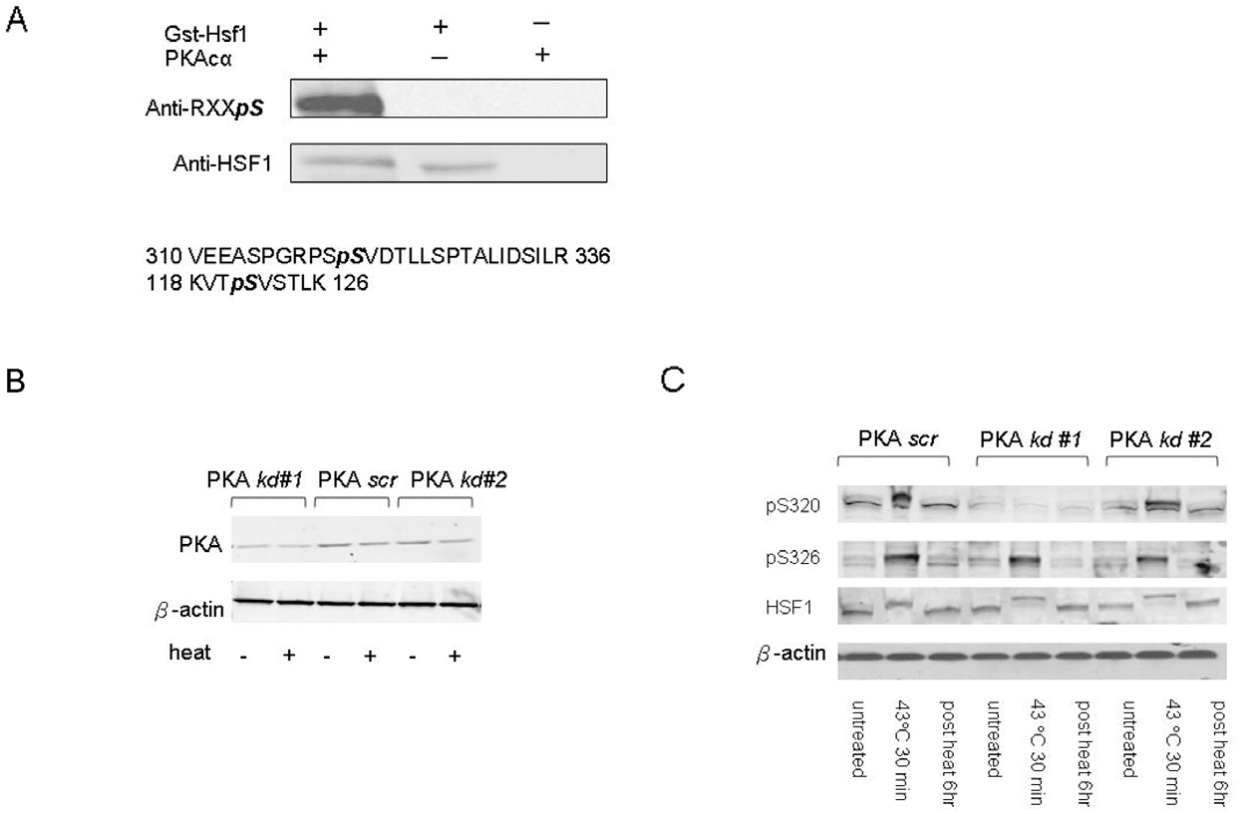
HSF1 is phosphorylated on serine 320 by PKAcα. *A*) Purified GST-tagged HSF1 was phosphorylated *in vitro* by PKAcα. Samples were then fractionated by 10% SDS-PAGE and initially immunoblotted for PKAcα phospho-substrate. The membranes were then stripped and re-blotted with anti-HSF1 antibodies. The identities of the PKAcα - modified phospho-peptides were determined by isolating the PKA-phosphorylated GST-HSF1 with 10% SDS-PAGE, trypsin digestion of the GST-HSF1 band and identification of peptides by LTQ-Orbitrap MS and database analysis. *B*) PKAcα expression in control (PKA *scr*) and PKA knockdown (PKA *kd,#1 and 2*) HeLa cells with or without heat shock. PKAcα levels with or without shRNA knockdown were measured by anti-PKAcα antibody immunoblot. *C*) Phosphorylation of HSF1 S320 and S326 in response to heat. Control (PKA *scr*) and PKAcα knockdown (PKA *kd#1 and 2*) HeLa cells were treated with or without heat shock as indicated. Phospho-HSF1 expression levels were then evaluated by western blot using anti-phospho-HSF1 antibodies (anti-pS320 and anti-pS326). β-actin expression was also measured as loading control and for quantitative normalization. Experiments (A–C) were carried out in duplicate with similar results.

We next investigated whether PKAcα could phosphorylate HSF1 when the two proteins were incubated together *in vitro*. Purified GST-HSF1 was mixed with purified PKAcα and ATP for a range of incubation times to maximize the extent of HSF1 phosphorylation. The reaction mixtures were then fractionated by SDS-PAGE and analyzed by immunoblot for HSF1 and for HSF1 phosphorylation on PKA consensus phosphorylation motif RXXpS, using anti-RXXpS antibodies (Fig. 2A). Abundantly phosphorylated samples of HSF1, detected by this antibody were then digested in-gel with trypsin and analyzed by mass spectrometry (Fig. 2A). Database analysis of the trypsin fragments indicated the generation of two principal phosphopeptide species, V310-R336 containing a potential site for PKAcα at S320 within a consensus PKA motif and K118-K126 containing S121 (Fig. 2A). We have shown that the latter site (S121) is, when phosphorylated, inhibitory for HSF1 under resting conditions. However, the influence of phospho-S121-HSF1 is overridden during heat shock under which conditions it does not influence transcription and becomes dephosphorylated [18]. We therefore concentrated on the novel S320 phosphorylation site. We examined the role of heat shock and PKAcα expression on HSF1-S320 phosphorylation using commercial antibodies specific for this phosphorylated form of HSF1 (Fig. 2B, C). Cells apparently contain significant background levels of phospho-S320-HSF1 and phosphorylation increased immediately after heat shock, before decaying back to basal levels by 2 hr recovery at 37°C, correlating well with the period of increased *trans*-activation by heat shock (Fig. 2C). We then carried out experiments in which intracellular PKAcα levels were reduced by stable knockdown with small hairpin RNA packaged in lentivirus (Fig. 2B). We employed two such shRNA species, PKAkd#1 and PKAkd#2, one of which (PKAkd#1) efficiently reduced PKAcα levels in infected cells while the other (PKAkd#2) was relatively less effective (Fig. 2B). Phospho-S320-HSF1 levels were significantly reduced in cells expressing PKAkd#1 compared to cells expressing PKAkd#2 or control cells infected with lentivirus enclosing a scrambled RNA species (PKA scr) (Fig. 2C). As a further control, we investigated HSF1 phosphorylation at the adjacent serine 326 using antibodies specific to this phosphorylated motif. HSF1 phosphorylation at this site was also increased by heat shock, although knockdown of PKAcα had minimal effects on S326 phosphorylation (Fig. 2C). Phosphorylation of HSF1 at these two adjacent serine residues, although induced with similar kinetics by heat shock, is evidently regulated independently. It is notable that HSF1 hyperphosphorylation was not markedly ablated by PKAcα knockdown conditions that led to reduced phospho-S320-HSF1 suggesting that other posttranslational modifications may contribute to the electrophoretic mobility shift seen in HSF1 after heat shock (Fig. 2 C).

### (3) Intracellular localization of HSF1 and PKAcα without and with stress

**Figure 3:**
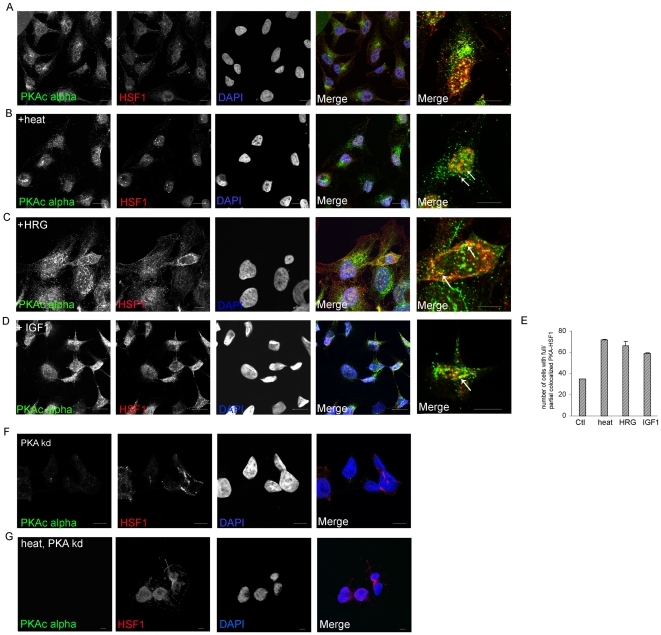
Intracellular location of PKAcα under HSF1 in different activation conditions. *A–D*) MCF-7 cells were plated and treated without and with heat shock at 43°C for 1 hour (A, B). Cells were also treated with 30 µM HRG (C) and 100 ng/ml IGF-1 (D) for 24 hours. Cells were then fixed and probed for PKAcα and HSF1 localization with anti- PKAcα and anti-HSF1 antibodies. Cells were later stained with fluorophore conjugated secondary antibodies goat anti-rabbit Alexa 488 (for PKAcα, green) and goat anti-rat Cy3 (for HSF1, red) antibodies. Nuclei were visualized with DAPI (nucleus, blue). In experiments A–D, co-localization is indicated with arrows in merge images *E*) Numbers of cells with partial or complete co-localization of HSF1 and PKAcα were counted for three independent experiments. The values for treated cells (heat, HRG, IGF1) are significantly different from non-treated control cells at p<0.001 (Student's t-test) under each condition. *F,G*) HeLa cells were infected with PKA shRNA (*kd #1*) and then incubated (G) at 43°C for 1 hour or sham heated but maintained at 37°C (F). Cells were stained with anti-PKAcα and anti-HSF1 antibodies. For indirect immunofluorescence, cells were stained with secondary antibodies; goat anti-Alexa 488 (PKAcα, green) and goat anti-rat Cy3 (for HSF1, red). Nuclear staining (DAPI) is also shown for these cells. Scale bar 5 µm. Experiments (A–D, F, G) were repeated at least 3 times with consistent findings.

We next examined the intracellular localization of HSF and PKAcα before and after stress using indirect immunofluorescence and confocal microscopy. In the absence of stress, PKAcα was found in punctuate *foci* in the perinuclear and nuclear regions of cells and HSF1 largely in the nucleus as described previously [24], [28] (Fig. 3A). After heat shock, HSF1 was also localized to nuclei and was concentrated in a number of punctuate foci (stress granules) also as observed previously (Fig. 3B). Co-localization or partial co-localization of the two proteins was observed in the nuclei in many cells after heat shock, although a large proportion of PKAcα remained in the perinuclear as well as in the cytosol in some cells (Fig. 3B). Quantitation by cell counting indicated that in approximately 30% of cells a proportion of the HSF1 was associated with PKAcα before heat shock, and this proportion increased to approximately 70% after heat shock (Fig. 3A, 3B, 3E). Heat shock did not however cause quantitative translocation of the PKAcα to the nucleus, suggesting that stable interaction after heat shock involves only a fraction of the cytosolic PKAcα or that perinuclear PKAcα may be capable of phosphorylating HSF1 in that region and help in translocation of HSF1 to the nucleus. Some cells showed nuclear localization of PKAcα and partial co-localization with HSF1 suggesting direct association between them as indicated in the earlier immunoprecipitation experiments. Not all PKAcα-containing punctate structures were associated with HSF1 in the nucleus, a finding which is not unexpected as nuclear PKAcα can associate with and phosphorylate other key components such as CREB (cyclic AMP responsive factor) after heat shock (A. Murshid & SK Calderwood, in preparation). Although HRG and IGF-1 activate transcription through HSF1, we did not observe the accumulation of the factor in stress granules after these treatments in most cells. However HSF1 association with PKAcα in nuclei was increased from 30% to approximately 60% after treatment with both HRG (Fig. 3C, 3E) and IGF-1 (Fig. 3D, 3E). (Stress granules appear to be involved in regulation of Satellite RNA during heat shock rather than HSP-encoding genes) [40].

We next examined a potential role for PKA in the nuclear localization of HSF1 after heat shock. In controls with unperturbed levels of PKAcα, HSF1 was mostly nuclear and after heat shock localized to stress granules in the nucleus (Fig. 3A, B) as described above [24], [41]. After PKAcα knockdown as in Fig. 2C, (PKA *kd #1*), the majority of the HSF1 was banished to punctate aggregates in the cytoplasm of both control cells and heat shocked cells (Fig. 3F, G). PKAcα levels were markedly reduced in these cells expressing the shRNA, as would be predicted (Fig. 3G). In control cells expressing the scrambled RNA sequence (PKA scr) HSF1 distributions in unheated and heat shocked cells were similar to those in wt cells indicating that the effects of PKA *kd #*1 are due to PKAcα knockdown rather than an artifact of the treatment conditions (Fig. S2, S3). Intracellular localizations of HSF1 in control and knockdown (PKA *kd #1)* cells were quantitated by cell counting and also shown in Fig. S3.

As previous studies suggest that HSF1 is regulated by nucleocytoplasmic shuttling and that nuclear export is an important component in this process, we next examined the potential role of the principal exportin, CRM1/exportin-1 in PKAcα-regulated localization of HSF1 under control and stress conditions. A role for CRM1 was examined using the inhibitor leptomycin-B (LMB). As described above, HSF1 was localized by immunofluorescence mainly in the nuclear compartments of cells with or without heat shock (Fig. 3A, B, Fig. S2, S3). We next examined whether LMB could reverse the inhibitory effects on HSF1 nuclear localization of PKAcα knockdown. Our hypothesis was that HSF1 phosphorylation by PKAcα might be required for nuclear accumulation of HSF1 by decreasing CRM1-mediated nuclear export. However, addition of LMB failed to reverse the effects of PKAcα knockdown on redistributing HSF1 to the cytoplasm in heat shocked cells (Fig. S2 D, E). Curiously, LMB was effective in permitting nuclear localization of HSF1 in unstressed cells even under conditions of PKAcα knockdown (Fig. S3). However, nuclear localization of HSF1 in stress conditions appears to be regulated in a PKAcα-dependent manner by an unconventional pathway that does not involve a major role for CRM1/exportin1. Indeed we were able to detect by co-immunoprecipitation analysis significant binding of CRM1 to HSF1 that was not modulated by heat shock (A Murshid & SK Calderwood, unpublished data).

**Figure 4:**
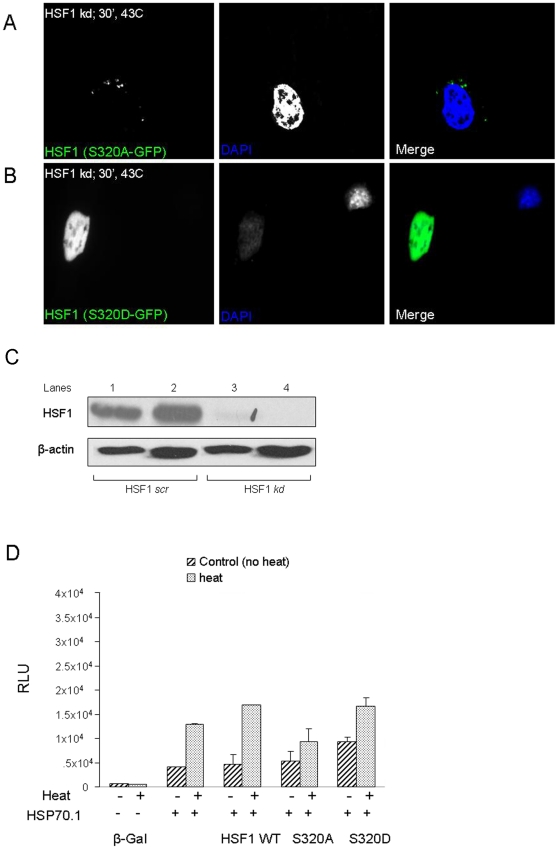
Phosphorylation at S320 influences the intracellular localization and activity of HSF1. *A, B*) Du145 cells with stable HSF1 knockdown were transiently transfected with either HSF1 (S320A)-GFP or HSF1 (S320D)-GFP and later heated at 43°C for 1 hour. Nuclei were detected using DAPI staining. *C*) Lysates from the HSF1 *kd* cells and control cells expressing scrambled RNA (*scr*) were analyzed in duplicate for HSF1 with anti-HSF1 antibodies. *D*) *Hsp70.1-Luc activity* with or without heat shock in untransfected cells, cells transfected with wtHSF1 or mutant HSF1. HeLa cells were transfected with plasmids encoding HSF1-GFP/HSF1, (S320A)-GFP/HSF1, (S320D)-GFP, β-gal, and pGL3-Hsp70-luciferase. Luciferase assay was performed in triplicate 24 hr following heat treatment and data expressed as mean luciferase level +/− SD. Comparisons between treatment groups were made using Student's t test. Experiments were repeated four times with similar results.

We then investigated the potential role of HSF1 phosphorylation at serine residue 320 in intracellular localization (Fig. 4). Mutant versions of GFP-tagged HSF1 were prepared in which S320 was changed either to aspartate (S320D) or alanine (S320A) and these constructs were then expressed in cells from a CMV-based promoter. S320A, being refractory to phosphorylation, would function as a null mutation while in S320D the negative charge of the carboxyl side chain of aspartate might mimic that of the phosphoserine. As the presence of wild-type HSF1 in such cells could complicate the interpretation, we carried these experiments in cells in which HSF1 was depleted by specific shRNA. We were unsuccessful in preparing stable HSF1 knockdown cell lines in either HeLa or MCF-7 as significant reductions in HSF1 were lethal to these cells (data not shown). We therefore used a prostate cancer cell line, DU-145 in which we were able to induce profound reduction in HSF1 levels by knockdown with shRNA and yet maintain viability. Inactivation of serine 320 by alanine substitution led to an HSF1 species refractory to heat-induced nuclear localization and which was detected in cytoplasmic foci (Fig. 4A). In cells expressing HSF1-GFP (S320D), this mutant form of HSF1 was localized to the nucleus after stress, indicating a role for negative charge at serine 320 in heat-induced nuclear localization of HSF1 (Fig. 4B). The predominantly nuclear localization of HSF1-GFP (S320D) (Fig. 4 B) resembled that of wt HSF1 after heat shock (Fig. 3 B, F), while the largely cytoplasmic localization of S320A-HSF1 (Fig. 4A, C) resembled localization of wt HSF1 after PKAcα knockdown by PKAkd#1 (Fig. 3F). These data support the hypothesis that PKAcα-mediated HSF1 phosphorylation at S320 plays a significant role in nuclear localization. To further determine the role of HSF1 phosphorylation at S320 in co-localization with PKAcα, we expressed FLAG-PKAcα in HeLa cells and then stained for phospho-S320 HSF1 in control, heat shocked (43°C, 1 hr) and HRG treated cells. We found similar patterns of partial co-localization of phospho-S320-HSF1 and PKAcα in perinuclear to nuclear regions as seen previously for total HSF1 in Fig. 3, (Fig. S4). HSF1 may thus dissociate from PKAcα but remain phosphorylated on S320.

We next examined the transcriptional activity of these HSF1-GFP mutants (Fig. 4D). These experiments were carried out in cells expressing wtHSF1 as we aimed to investigate a potential dominant negative role for the null mutant (HSF1-S320A). Exposure to heat shock led to activation of the *hsp70.1*-luciferase promoter-reporter construct (*hsp70*.1-Luc) in untransfected cells and such activity was increased in cells transfected with wild-type HSF1 (Fig. 4D). However, after HSF1-S320A transfection, heat shock induced *hsp70.1*-Luc activity was significantly (p<0.01) decreased below the levels seen in untransfected cells suggesting potential “dominant negative” effects of S320A in quenching activation of the endogenous HSF1 by heat shock. Expression of HSF1-S320D amplified the level of heat shock-induced luciferase activity by a similar magnitude compared to activation by wild-type HSF1 (Fig. 4D). Interestingly, cells expressing HSF1-S320D had significantly elevated mean luciferase levels (p<0.05) even in non-heat conditions suggesting that gain of negative charge at S320 may mediate the activating effects of heat shock (Fig. 4D).

### (4) Regulatory role for PKAcα in stress-mediated localization of HSF1 to the *hsp70.1* promoter and in *hsp70.1* transcription

**Figure 5:**
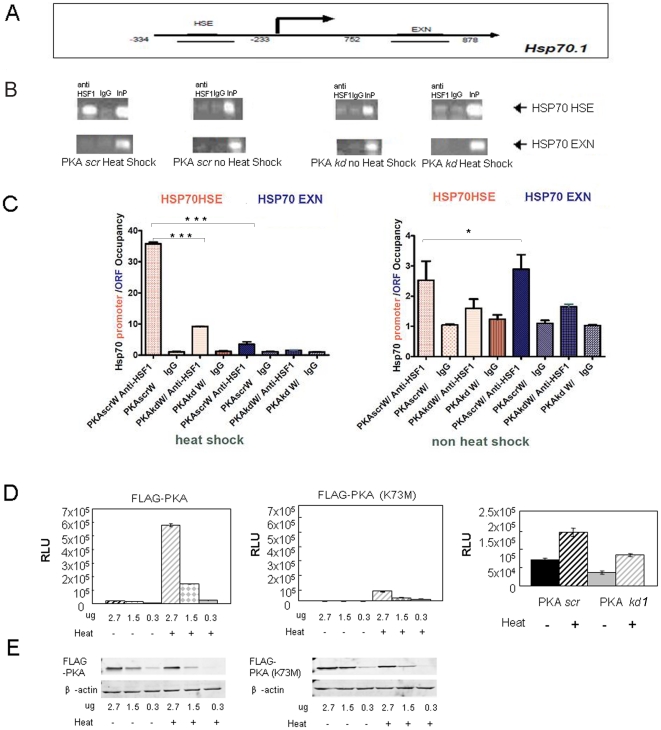
PKAcα knockdown inhibits HSF1 association with the *hsp70.1* promoter. (A) Diagram of primers mapping to the *hsp70.1* gene. *B*) ChIP experiments. HeLa cells either with PKA knockdown (PKA *kd #1*) or expressing a scrambled control RNA (PKA *scr*) were incubated without or with heat shock (43°C, 1 hr). Cells were then fixed with formaldehyde, sonicated and analyzed by ChIP assay using HSF1 antibodies or preimmune-IgG as control. The immunoprecipitated DNA was PCR amplified using the primers specific to the promoter (HSE, upper lane) or ORF (EXN, bottom lane) sequences in *hsp70.1*. Each panel (from left to right) shows the PCR products from the HSF1 ChIP assay (anti-HSF1), IgG ChIP or from control sample of genomic DNA (input). Experiments were performed three times with similar findings. *C*) ChIP PCR products were also analyzed using the ABI 7300 real time PCR system and the fold increase by 2^−ΔΔCT^ method. Levels of HSF1 ChIP PCR products were compared with the products associated with pre-immune-IgG and plotted for the respective regions of *hsp70.1*. ChIP experiments were each performed at least 4 times with and data plotted as mean + SD with consistent findings. Differences in gene occupation by HSF under the different conditions were analyzed statistically by Student's t test. Promoter occupancy by HSF1 after heat shock was significantly higher than in PKAcα knockdown cells or occupancy of Hsp70EXN regions with or without PKAcα and significance is indicated: *** p<0.001 (left panel). Differences in HSF1 binding to promoter (HSP70HSE) or ORF regions (HSP70EXN) in non-heat shocked cells were not significant (p>0.05). *D*) Luciferase assay of *Hsp70.1* promoter-reporter activation in response to heat shock. HeLa cells were transfected with plasmids expressing either wild type PKAcα (FLAG-PKA) or kinase dead PKAcα (FLAG-PKA K73M). The amounts of plasmid transfected under each condition are indicated on the abscissa. We also measured *hsp70*.1-Luc activity in cells without or with heat shock both under control conditions in cells expressing scrambled RNA (PKA *scr*) or after PKAcα knockdown (PKA *kd #1*). Luciferase assays were then performed in triplicate following heat treatment and activity plotted as mean +/− standard deviation. Statistical significance of differences in *hsp70*.1-Luc activity under the various conditions was compared by Student's t test. Experiments were performed three times with similar findings. (E) Anti-FLAG immunoblot analysis was carried out to demonstrate the relative levels of intracellular PKAcα expression achieved under these transfection conditions and are shown beneath the luciferase panels. β-actin expression under these conditions is shown as loading control and was used for quantitative normalization. Experiments were carried out 3–5 times with consistent findings.

We next examined whether PKAcα is required for HSF1 binding to the *hsp70.1* gene *in vivo* using ChIP analysis (Fig. 5). We measured association of native HSF1 with *hsp70.1* chromatin using anti-HSF1 antibodies. Potential HSF1 association with two sequences in *hsp70.1* chromatin was investigated, including a region within the promoter containing the most proximal heat shock element (hsp70HSE) and a downstream sequence in the open reading frame (hsp70EXN) (Fig. 5A).

Fig. 5B shows ChIP analysis indicating HSF1 binding to *hsp70.1* chromatin. In each panel we show PCR analysis of DNA associated with HSF1 in the first lane, a control ChIP analysis with pre-immune IgG in the second lane, as well as an indication of the input in the third lane. Experiments are carried out without and with heat shock in cells treated with the shRNA PKAkd#1 described above (PKAshRNA) or in controls expressing a scrambled RNA sequence (PKA*scr*). Using this approach we detected HSF1 binding to the HSE region of *hsp70.1* after heat shock in PKA*scr* (Fig. 5B top left panel). HSF1 was not detected in association with the EXN region of *hsp70.1* in any of the conditions and did not appear to bind Hsp70HSE in the absence of heat shock or after PKAcα knockdown with or without heat shock (Fig. 5B). These experiments were repeated using quantitative real time RT-PCR and are shown in Fig. 5C. Heat shock led to an approximate 35-fold enrichment of HSF1 on the HSE region and such binding was inhibited in cells after PKAcα knockdown (PKAkd#1) (Fig. 5C). Minimal binding of HSF1 to the HSE was seen in PKAshRNA cells and in the absence of heat shock (Fig. 5C). Minimal association of HSF1 with HSP70 EXN was seen in under each of the conditions (Fig. 5C).

We next examined the requirement for PKAcα in transcriptional activation of HSF1 by heat shock. Modulation of PKAcα activity by overexpression or knockdown influenced markedly the activity of the *hsp70.1*-Luc reporter (Fig. 5). Increasing intracellular PKAcα levels by forced expression from a viral promoter (pCMV.FLAG-PKAcα) led to a significant (p<0.005) plasmid dose-dependent increase in heat-induced *hsp70.1*-Luc activity [36] (Fig. 5D (left panel). The increased levels of PKAcα expression in these experiments are indicated in the anti-FLAG antibody immunoblot shown in the lower part of the figure and bands are quantitated (in Fig. 5E, bottom). The *hsp70.1* promoter was not markedly activated by forced expression of a kinase dead PKAcα construct (from transfected pCMV.FLAG-PKAcα-K73M), indicating a requirement for the catalytic activity of PKA in enhanced HSF1 activation (Fig. 5D, middle panel).

We next examined the effect of reducing PKAcα levels by RNA interference (using the shRNA construct PKAkd#1) on hsp70.1-Luc activity. Heat shock activated *hsp70.1*-Luc in control cells expressing scrambled RNA (PKAscr), while PKAcα knockdown significantly (p<0.05) reduced both basal and heat-induced *hsp70.1*-Luc activity (Fig. 5D, right panel). The relative levels of PKAcα expressed in cells without and with knockdown by PKAshRNA are indicated in the earlier figure (2 B).

## Discussion

Our experiments therefore suggest a novel mechanism in the activation of hsp70 after stress, involving HSF1 association with PKAcα and phosphorylation by this kinase at a novel site (serine 320) (Fig. 1). These conclusions are confluent with earlier studies suggesting a role for PKA in activation of an *hsp70* promoter [42]. The mechanisms dictating PKAcα binding to HSF1 in stress are not entirely clear but do not appear to involve major roles for adenylate cyclase or cAMP (Fig. 1D). We observed only a minor amount of binding of the regulatory R1 domain of PKA to HSF1 (Fig. 1). It seems likely that activation of phosphorylation by the bound PKAcα involves the transition in HSF1 structure that occurs after heat shock, from a compacted form, constrained by intramolecular coiled-coil and molecular chaperone interactions to a more expansive conformation [43], [44], [45], [46], [47]. Such a transition may expose domains previously cryptic within the inactive protein. We also observed a basal level of HSF1- PKAcα binding and S320 phosphorylation in HeLa and MCF-7 cells (Fig. 1, 2). This may reflect the fact that HSF1 possesses basal transcriptional activity in many human malignant cells lines and is required for survival in these cells [1], [2], [48]. Previous studies also showed that, when HSF1 is partially activated to a more relaxed, DNA binding form by exposure of cells to salicylate or menadione, it can then be rendered transcriptionally competent by exposure of cells to protein phosphatase inhibitors [14]. In addition our *in vitro* studies showed a similar two part activation and although ATP is not required for the formation of HSF1 trimers and for DNA binding it is essential for full transcriptional activation. Serine 320 is within the central region of HSF1 that is regulated by heat shock independently of trimerization or DNA binding [49], [50]. The regulatory domain of HSF1 is repressive to transcription under normal temperatures while stress not only overturns repression but leads to *trans*-activation considerably greater in magnitude than might be accounted for by reversal of repression [49], [50]. The original studies suggested that regulatory function is bounded by amino acids 221–310 although the discovery of activating phosphorylation at serines 320 and 326 suggests extending the domain to at least serine 326 (Fig. 2) [21], [49], [50]. The regulatory domain of HSF1 could function as a signaling platform in response to protein kinases as it contains multiple serine and threonine residues [16], [51]. This region contains serines 303 and 307 that are repressive when phosphorylated and could exert some of the inhibitory functions of the regulatory domain seen at normal temperatures. Inhibition of *trans* activation exerted through this region appears to involve a pathway in which dual phosphorylation of HSF1 at serines 307 and 303 by ERK1 and GSK3, respectively leads to nuclear export of HSF1 through a mechanism involving recruitment of 14-3-3 family members, thus removing HSF1 from *HSP* promoters [20], [24], [26], [52]. In addition an adjacent residue at lysine 298 can be sumoylated in a mechanism also dependent on serine 303 phosphorylation [27], [53]. These inhibitory posttranslational modifications can be overridden in heat shocked cells even though serine 303 remains abundantly phosphorylated, 14-3-3 remains bound and K298 is sumoylated [28]. The central regulatory region also contains positively acting phosphorylation sites at serines 230, 326 and, as shown here at S320 (Fig. 2) [19], [21]. It is not clear how posttranslational modifications in this domain during stress might override the inhibitory influence of S303 phosphorylation and stimulate HSF1 *trans* activation. However, the current studies suggest a role for PKAcα and S320 in permitting HSF1 to accumulate in the nucleus and in nuclear stress granules, bind to the *hsp70.1* promoter and activate *hsp70.1* transcription (Figs. 3, 4, 5). PKAcα phosphorylation of S320 may be involved in the accumulation in nuclei by reversing of nuclear export [28]. The exact mechanism involved is, however not clear. Nuclear localization depends on relative rates of nuclear import and nuclear export. Human HSF1 contains bipartite nuclear localization signals (NLS) adjacent to leucine zipper domains, N-terminal to the regulatory domain [54]. Earlier studies showed that nuclear import involving one or more of these NLS sequences occurs continuously with or without stress and that nuclear accumulation is most likely due to blockade of nuclear export [55]. A role for reversible binding of HSF1 to the major nuclear export protein CRM1/exportin1 in stress seems unlikely as this protein binds avidly to HSF1 with or without heat shock (A. Murshid & S.K. Calderwood, unpublished data). It is also notable that depletion of PKAcα did not completely inhibit HSF1 (Fig. 5 D). This may reflect the incomplete reduction in PKAcα levels after expression of shRNA in the cells (Fig. 2 C). It seems however likely that other posttranslational modifications in the regulatory domain also contribute to HSF1 activation, notably phosphorylation at serines 230 and 326 [21], [27]. One suggested mechanism for differential regulation of HSF1 in stressed and unstressed conditions is that an altered phosphorylation landscape in the central regulatory domain may permit a switch between activator and inhibitor activity. This region of HSF1, rich in proline and serine residues, would be predicted to lack secondary structure motifs and phosphorylation could impose alternative structures due to changes in charge distribution. In addition, such phosphorylation patterns might also form binding sites for (as yet undetected) regulatory proteins.

We did not examine here the potential effects of HSF1 binding on the kinase activity of PKA. However, one recent study indicates that HSF1 may be involved in upstream activation of PKA during cell transformation [1]. Although we did not study HSF1 mediation of intracellular PKA activity, our studies do indicate sturdy interaction of HSF1 and PKAcα after treatment with transforming growth factor HRG (Figs. 1, 2). Previously we showed that, HRG directly activates HSF1 and that HSF1 mediates some of its tumorigenic effects [56].

In conclusion therefore, we have demonstrated physical and functional interaction between protein kinase A and HSF1. The PKA catalytic subunit PKAcα binds and phosphorylates HSF1 on serine 320, a modification essential for nuclear localization, association with the *hsp70.1* promoter and transcription. A role for PKAcα in the network of signaling events leading to the activation of HSF1 during the stress response is suggested.

## Supporting Information

Figure S1 HSF1 interacts with PKAcα. HeLa proteins adsorbed to GST (control) or GST-HSF1 were eluted from GSH-4B beads by reduced glutathione, and proteins analyzed by SDS-PAGE and silver staining. Silver-stained bands were digested in-gel with trypsin and eluted peptides analyzed by matrix-assisted laser desorption/ionization-time of flight-mass spectroscopy using a Voyager DE-PRO (Applied Bio-systems) and proteins identified by mass fingerprinting using database (SwissProt.8.17.2002). Peptides (AKEDFLK, VMLVKHK, QIEHTLNRK) with an exact match to murine (8.66), rat (8.64), human (8.65) PKAcα and bovine PKAcβ (8.65) were found. (MOWSE scores are shown in parenthesis.) Mass spectrometry data were confirmed by immunoblot assay shown here carried out here on eluates from either unmodified GST or GST-HSF1, using anti-PKAc antibodies. Experiments were performed in duplicate with similar findings.
(0.04 MB TIF)

Figure S2 Effects of LMB on localization of HSF1 with or without PKAcα knockdown. A–D) HeLa cells were treated without or with LMB for 16 hours. Cells were later probed for PKAcα and HSF1 with anti-PKAcα antibodies and anti-HSF1 antibodies respectively. Cells were later stained with second antibodies goat anti-rabbit Alexa 488 (for PKAcα, green) and goat anti-rat Cy3 (for HSF1, red). Nuclei were visualized using DAPI (blue) staining. C, D, E) HeLa (PKAscr) and HeLa (PKAkd#1) cells were treated with LMB for 16 hours before heat shock (43oC, 1 hour) and fixed cells were later stained for PKAcα and HSF1 with anti- PKAcα and anti-HSF1 antibodies as described in A. E) HeLa (PKAkd#1) cells heat shocked (43oC, 1 hour) or not. Cells were then stained for PKAcα and HSF1 using the antibody cascades employed in A–D. All experiments were repeated at least once with consistent findings.
(4.76 MB TIF)

Figure S3 Quantitative analysis of the distribution of HSF1 in wt HeLa, HeLa (PKA scr) and HeLa (PKA kd#1) cells under non-treated, heat shock or LMB treated conditions as indicated in figure was performed after confocal fluorescence microscopy. The sub-cellular distribution of HSF1 was scored according to whether the protein was detected in the nucleus, in both nucleus and cytoplasm or strictly in the cytoplasm.
(0.13 MB TIF)

Figure S4 PKAcα associates with pS320 HSF1 in different activation conditions. A) MCF7 cells were transfected with FLAG- PKAcα for 22 hr. Cells were then incubated at 43oC for 1 hr, fixed and stained for FLAG and pS320 with mouse monoclonal anti-FLAG-ab (red, Cy3-secondary ab) and Rabbit polyclonal anti pS320-ab (green, Alexa 488-secondary ab). B) MCF 7 cells were transfected with FLAG- PKAcα for 22 hr, fixed and stained as in A. C) MCF7 cells were transfected with FLAG- PKAcα for 22 hr. Cells were treated with HRG (30 µM HRG for 24 hr) before fixation. Fixed cells were stained as in A. Experiments were performed three times with consistent findings.
(1.09 MB TIF)
