# Correction: Protein Kinase A Binds and Activates Heat Shock Factor 1

**DOI:** 10.1371/annotation/5879464d-8556-4c3e-b11c-a96cbbff44a6

**Published:** 2010-11-17

**Authors:** Ayesha Murshid, Shiuh-Dih Chou, Thomas Prince, Yue Zhang, Ajit Bharti, Stuart K. Calderwood

The first four authors, Ayesha Murshid, Shiuh-Dih Chou, Thomas Prince, and Yue Zhang, should be noted as contributing equally to this work.

