# Supplementary figures and images for: Protein Kinase A Binds and Activates Heat Shock Factor 1
Source: PLoS One. 2010 Nov 9;5(11):e13830. doi: 10.1371/journal.pone.0013830 (PMC2976705; doi:10.1371/journal.pone.0013830)

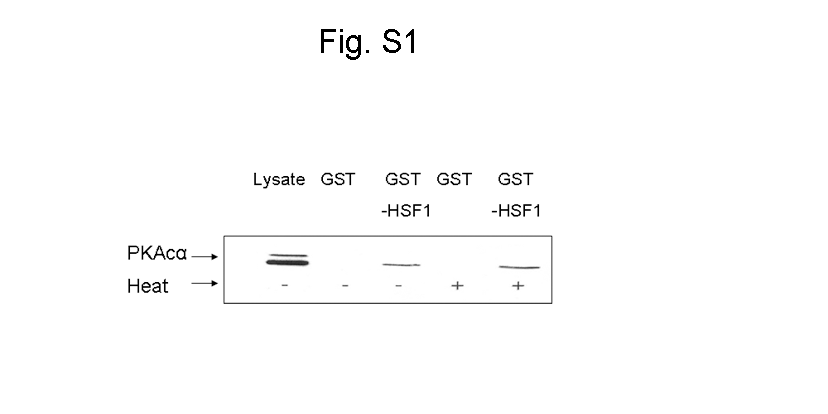

Supplement: Figure S1 — HSF1 interacts with PKAcα. HeLa proteins adsorbed to GST (control) or GST-HSF1 were eluted from GSH-4B beads by reduced glutathione, and proteins analyzed by SDS-PAGE and silver staining. Silver-stained bands were digested in-gel with trypsin and eluted peptides analyzed by matrix-assisted laser desorption/ionization-time of flight-mass spectroscopy using a Voyager DE-PRO (Applied Bio-systems) and proteins identified by mass fingerprinting using database (SwissProt.8.17.2002). Peptides (AKEDFLK, VMLVKHK, QIEHTLNRK) with an exact match to murine (8.66), rat (8.64), human (8.65) PKAcα and bovine PKAcβ (8.65) were found. (MOWSE scores are shown in parenthesis.) Mass spectrometry data were confirmed by immunoblot assay shown here carried out here on eluates from either unmodified GST or GST-HSF1, using anti-PKAc antibodies. Experiments were performed in duplicate with similar findings. (0.04 MB TIF) [file pone.0013830.s001.tif]

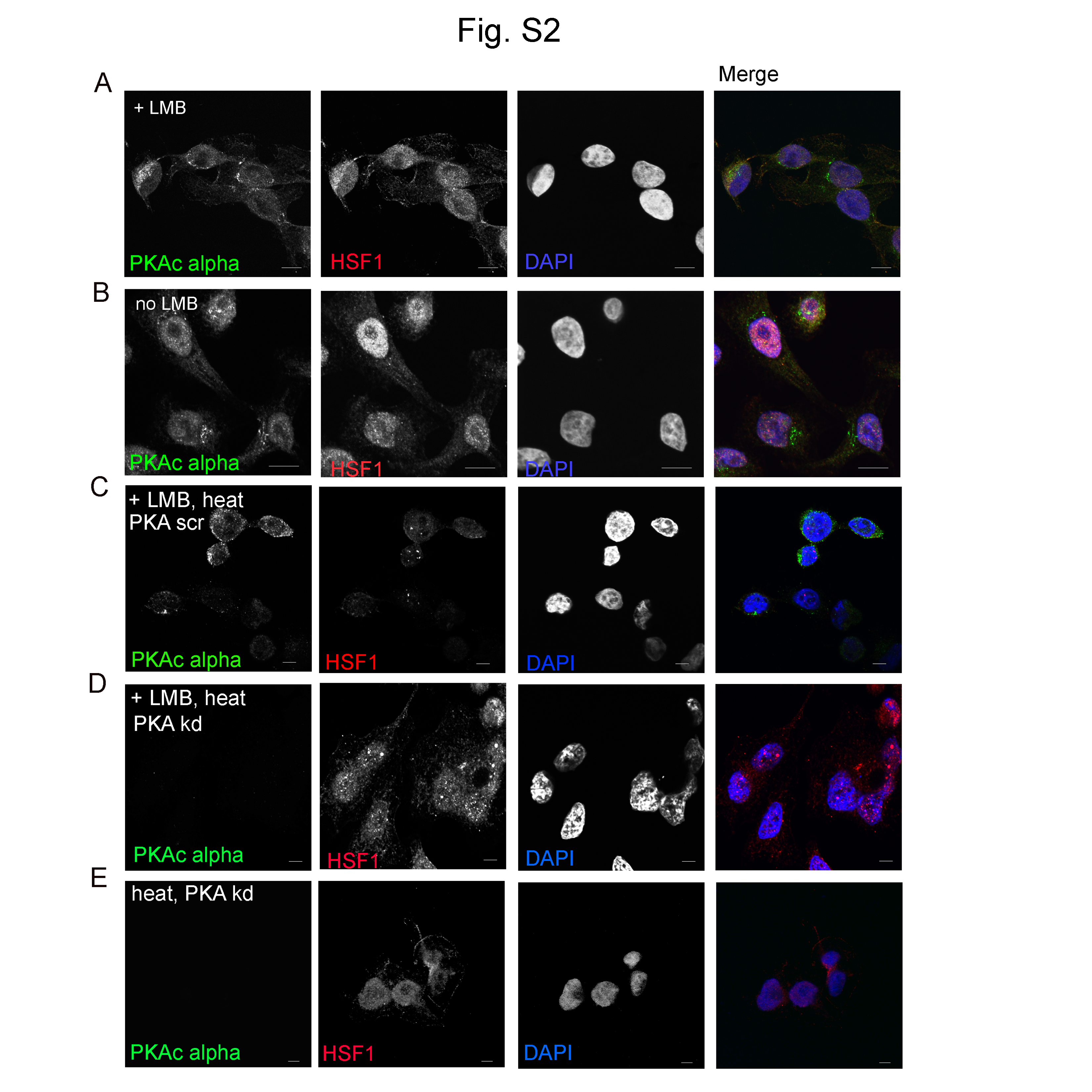

Supplement: Figure S2 — Effects of LMB on localization of HSF1 with or without PKAcα knockdown. A–D) HeLa cells were treated without or with LMB for 16 hours. Cells were later probed for PKAcα and HSF1 with anti-PKAcα antibodies and anti-HSF1 antibodies respectively. Cells were later stained with second antibodies goat anti-rabbit Alexa 488 (for PKAcα, green) and goat anti-rat Cy3 (for HSF1, red). Nuclei were visualized using DAPI (blue) staining. C, D, E) HeLa (PKAscr) and HeLa (PKAkd#1) cells were treated with LMB for 16 hours before heat shock (43oC, 1 hour) and fixed cells were later stained for PKAcα and HSF1 with anti- PKAcα and anti-HSF1 antibodies as described in A. E) HeLa (PKAkd#1) cells heat shocked (43oC, 1 hour) or not. Cells were then stained for PKAcα and HSF1 using the antibody cascades employed in A–D. All experiments were repeated at least once with consistent findings. (4.76 MB TIF) [file pone.0013830.s002.tif]

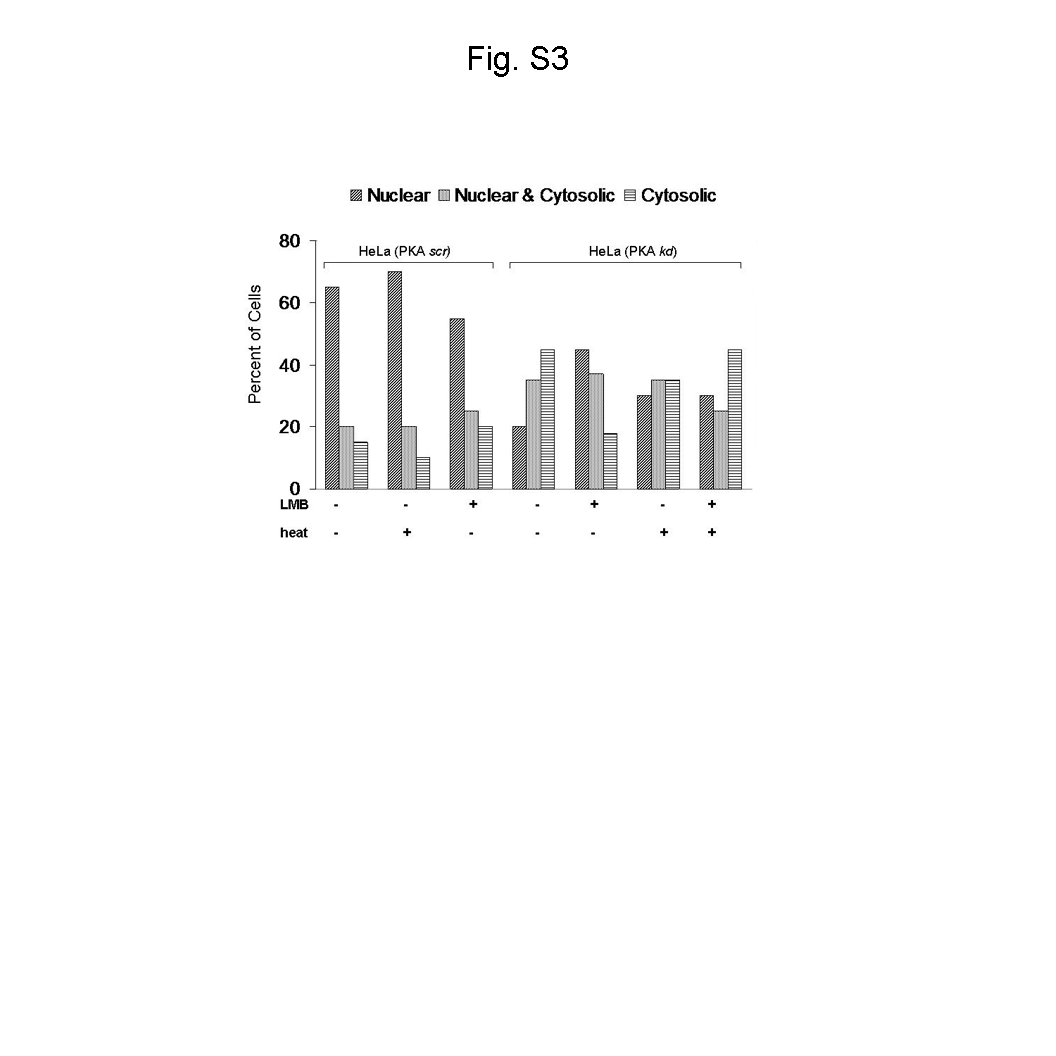

Supplement: Figure S3 — Quantitative analysis of the distribution of HSF1 in wt HeLa, HeLa (PKA scr) and HeLa (PKA kd#1) cells under non-treated, heat shock or LMB treated conditions as indicated in figure was performed after confocal fluorescence microscopy. The sub-cellular distribution of HSF1 was scored according to whether the protein was detected in the nucleus, in both nucleus and cytoplasm or strictly in the cytoplasm. (0.13 MB TIF) [file pone.0013830.s003.tif]

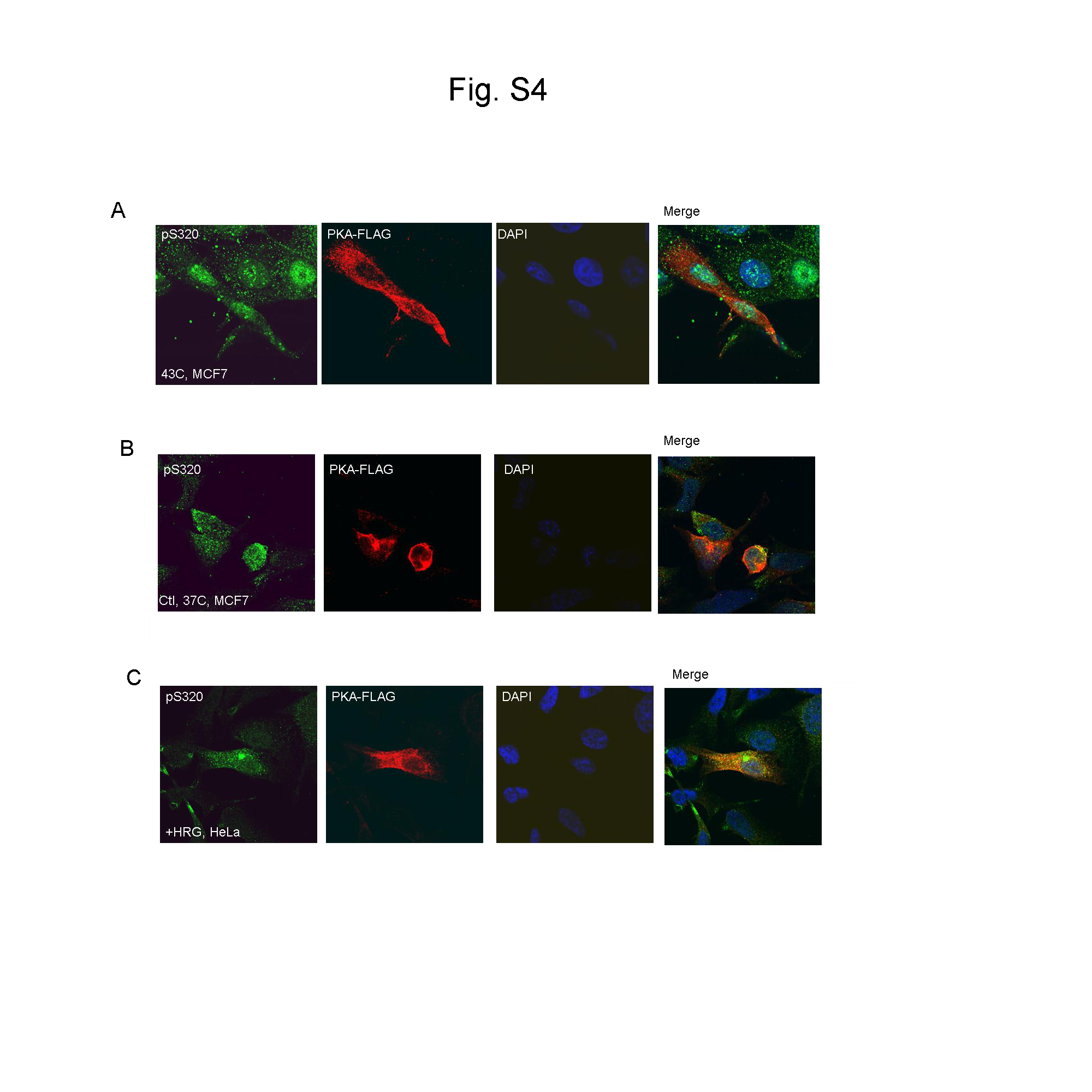

Supplement: Figure S4 — PKAcα associates with pS320 HSF1 in different activation conditions. A) MCF7 cells were transfected with FLAG- PKAcα for 22 hr. Cells were then incubated at 43oC for 1 hr, fixed and stained for FLAG and pS320 with mouse monoclonal anti-FLAG-ab (red, Cy3-secondary ab) and Rabbit polyclonal anti pS320-ab (green, Alexa 488-secondary ab). B) MCF 7 cells were transfected with FLAG- PKAcα for 22 hr, fixed and stained as in A. C) MCF7 cells were transfected with FLAG- PKAcα for 22 hr. Cells were treated with HRG (30 µM HRG for 24 hr) before fixation. Fixed cells were stained as in A. Experiments were performed three times with consistent findings. (1.09 MB TIF) [file pone.0013830.s004.tif]
